# Toward Intelligent Hemodynamic Monitoring: A Functional Approach

**DOI:** 10.1155/2012/630828

**Published:** 2011-07-26

**Authors:** Pierre Squara, Carl Waldmann

**Affiliations:** ^1^Intensive Care Unit, Clinique Ambroise Paré, 27 Bd Victor Hugo, 92200 Neuilly-sur-Seine, France; ^2^Intensive Care Unit, Royal Berkshire Hospital, London Road, Reading RG1 5AN, UK

## Abstract

Technology is now available to allow a complete haemodynamic analysis; however this is only used in a small proportion of patients and seems to occur when the medical staff have the time and inclination. As a result of this, significant delays occur between an event, its diagnosis and therefore, any treatment required. We can speculate that we should be able to collect enough real time information to make a complete, real time, haemodynamic diagnosis in all critically ill patients. This article advocates for “intelligent haemodynamic monitoring”. Following the steps of a functional analysis, we answered six basic questions. (1) What is the actual best theoretical model for describing haemodynamic disorders? (2) What are the needed and necessary input/output data for describing this model? (3) What are the specific quality criteria and tolerances for collecting each input variable? (4) Based on these criteria, what are the validated available technologies for monitoring each input variable, continuously, real time, and if possible non-invasively? (5) How can we integrate all the needed reliably monitored input variables into the same system for continuously describing the global haemodynamic model? (6) Is it possible to implement this global model into intelligent programs that are able to differentiate clinically relevant changes as opposed to artificial changes and to display intelligent messages and/or diagnoses?

## 1. Introduction

Thirty years ago, tracking the heart rate (HR) was the only means of automatic, continuous, real-time and noninvasive, hemodynamic monitoring. A more elaborate level of monitoring was necessarily invasive and required a central venous catheter for continuous pressure (CVP) assessment. A third level was based on the placement of a pulmonary artery catheter (PAC) and of an arterial line for continuous pulmonary artery pressure (PAP) and systemic arterial pressure (SAP) curve recording. 

From this traditional data monitoring, a complete haemodynamic diagnosis was obtained on demand by the measurement of a set of additional variables, such as cardiac output (CO), pulmonary wedge pressure (PWP), blood lactate, haemoglobin concentration (Hb), arterial haemoglobin oxygen saturation (SaO_2_), and mixed venous haemoglobin oxygen saturation (SvO_2_). From these elementary data, several other variables were derived such as pulmonary and systemic resistance to flow (PVR and SVR), right and left ventricles stoke work (RVSW and LVSW), and tissue oxygenation indices: oxygen arterial and venous content (CaO_2_ and CvO_2_), oxygen delivery (DO_2_), arteriovenous oxygen differences (AVD), tissue oxygen extraction (EO_2_), and oxygen consumption (VO_2_). 

Therefore a complete haemodynamic analysis was done only in a small proportion of patients, and when the medical staff had the time and inclination. This resulted sometimes in significant delays between an event and its diagnosis and therefore any treatment required.

Today, more haemodynamic variables are monitored continuously by less invasive means than before; for instance using pulse contour CO, as compared to traditional invasive discontinuous bolus thermodilution. We can speculate that we are now able to collect enough real-time information continuously in order to make a complete, real-time, haemodynamic diagnosis. It implies not only continuous data recording but also continuous analysis using artificial intelligence. This is what we call “intelligent haemodynamic monitoring”. In the engineering industry, a functional analysis system technique (FAST) is used in the conception and the development of a product. It would be helpful to clinicians if they share with engineers the same fundamental approach. This is a prerequisite for reinforcing the way industry and clinicians are collaborating for the benefit of our patients. Basically, we must answer three questions: when, why, how?

*When* is easy: continuously, real time. 

*Why?* Because patients are in a severely compromised state, there is sudden and large variability in their cardiovascular status and we speculate that continuous, real-time, monitoring with appropriate alarm settings will minimize the delay between an event and its appropriate treatment, leading to better outcome.

*How*, is the most difficult question to answer. We suggest a functional analysis as follows.

- What is the actual best theoretical model for describing haemodynamic disorders?
- What are the needed and necessary input/output variables for describing this model?
- What are the specific quality criteria and tolerances for collecting each input variable?
- Based on these criteria, what are the validated available technologies for monitoring each input variable, continuously, real time, and if possible non-invasively?
- How can we integrate all the needed reliably monitored input variables into the same system for continuously describing the global haemodynamic model?
- Is it possible to implement this reliably described haemodynamic model into intelligent programs able to differentiate clinically relevant changes versus artificial changes and to display intelligent messages and/or diagnoses?

## 2. Functional Approach

### 2.1. What Is the Actual Best Model for Describing Hemodynamic Disorders

The semantic frames we are using today for classifying haemodynamic disorders are still derived from the discontinuous measurements available early in critical care medicine development. From normal or abnormal ranges of CVP, PCWP, CO, and SAP, we are still defining diagnostic terms such as hypovolaemic, cardiogenic, obstructive or hyperdynamic shock from which we infer specific therapeutic interventions. However, these diagnostic categories are simplistic, lead to a loss of information, and may not trigger all necessary treatment. Integrating new continuously monitored variables is an opportunity to update our reasoning processes, based on more robust models of haemodynamic disorders. Doing this, we must avoid falling from Charybdis into Scylla and distorting the haemodynamic reality in order to fit in with available electronic languages or with new artificial variables developed by the industry in their efforts to find complete solutions by the use of one single technology.

The best theoretical model of shock for monitoring purposes would be that one giving a complete and comprehensive description of all possible pathological processes observed in clinical practice and for which evidence is provided. At this level of our analysis, we must only consider the physiological principles. Practical limitations will be considered afterwards, in an appropriate step of the functional analysis.

Within this scope, a haemodynamic physiologic and pathologic model has been developed for a complete computerized haemodynamic diagnosis in the 1990's [1, 2]. In this model, all mechanisms of shock are supposed to stem from tissue oxygen demand outstripping oxygen supply. A global oxygen consumption (VO_2_) below the needs (^*n*^VO_2_) is consequently the best way to describe a macrocirculation disorder [3, 4]. This model, established by a panel of experts [2] from basic physiology, has been validated by the evidence that modelled variables have the highest prognostic value as compared to other traditional elementary and derived variables [1]. In addition, the diagnostic categorizations based on this model have been shown to be at least equivalent to that of experienced intensivists [2, 5]. We can also consider that studies showing an improved outcome when DO_2_ was rapidly increased to cover estimated needs is indirect proof of validity of this model [6]. The principles are as follows. Practical examples can be found and/or created online (http://www.hemodyn.com/). 

As seen above, shock is defined by

(1)   VO2<VO2  nor  VO2VO2n<1.

Since VO_2_ = CO × AVD, we can estimate the required values of CO and AVD for reaching a given value of ^*n*^VO_2_, for each specific patient, at a specific moment in time. So now, we can express this as

(2)   VO2n=COn×AVDn.

Then (1) can be reformulated as

(3)   VO2VO2n={COCOn}×{AVDAVDn}.

Since DO_2_ = CO × CaO_2_ and EO_2_ = AVD/CaO_2_, the formula (3) can also be formulated as

(2 bis) VO2VO2n={DO2DO2n}×{EO2EO2n},

where ^*n*^DO_2_ and ^*n*^EO_2_ are the needed values of DO_2_ and EO_2_ to reach ^*n*^VO_2_

**Figure 1:**
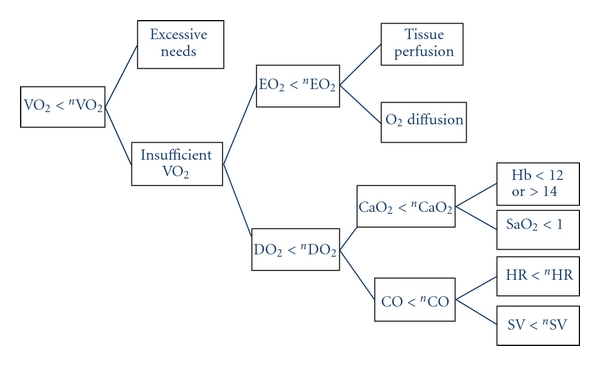
Root algorithm for representing the global hemodynamic model. The model gives priority to the higher box as compared to the lower. For example at the first step, the algorithm recommend decreasing excessive needs before looking at improving VO_2_.

From (3) we can see that shock, defined by VO_2_ < ^*n*^VO_2_, can be the result of a circulatory disorder (CO < ^*n*^CO) or a tissue disorder (AVD < ^*n*^AVD), or both, or insufficient compensation of one arm by the other. Since CaO_2_ is derived schematically by Hb×1.34×SaO_2_, it is immediately clear from (2 bis) that tissue hypoxia may occur due to (1) excessive demand and/or (2) insufficient CO, (3) insufficient SaO_2_, (4) insufficient Hb, and (5) insufficient EO_2_ (Figure 1). Therefore, the traditional clinical diagnostic categorisation of shock that referred mainly to mechanisms 2, 4, and 5, appear artificial and incomplete.

**Figure 2:**
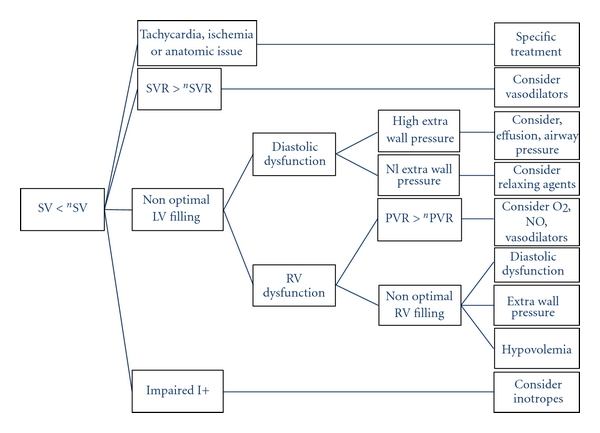
Subsequent algorithm dedicated to SV analysis. The needed SVR is that specific value of SVR allowing the generation of the minimum blood pressure (usually set at a mean value of 65 mmHg) with the needed CO. Similarly for needed PVR.

From this initial root, the model may be enriched as seen in Figure 2, based on the same principles, by comparing an actual value with a needed or minimum value for reaching adequate VO_2_ to cover needs. 

In this model, there is no normal or abnormal variable range. An adequate value is that one that allows compensation for the insufficiency of one or several other variables. Adequate limits can be eventually introduced into the model if other pathophysiological processes are involved. For example, there is no theoretical risk in decreasing EO_2_ until needed EO_2_ is reached while increasing CO or Hb up to very high values may be limited by myocardial ischemia or rheological impairment.

Following the initial root (Figure 1), the various mechanisms of shock may be subsequently analysed. Figure 2 continues the root algorithm when SV < ^*n*^SV (bottom right box of the Figure 1). Similarly an algorithm is described for each box of Figure 1. The basic principles are maintained in the secondary algorithms.

In Figure 2 we can see that the priority (higher lines) is still given to metabolic equilibrium at lower cost. First by restoring the coronary flow if necessary, then by decreasing the metabolic demand, finally by increasing the cardiac power first by filling, then if really necessary by inotropic support. 

At each step (boxes in the figures), the best variables for answering the question and reaching the objective can be updated according to any recent developments and to the state of the art. For example, in Figure 1, tissue perfusion can be analysed from diuresis, brain activity, the StO_2_, the local capillary flow, or nay other indicator to be invented. In Figure 2, right ventricle (RV) filling may be analysed by monitoring and targeting adequate values of right atrial pressure (RAP), RV end diastolic volume, pulmonary pulse pressure variation, or any other validated variable. Therefore, at each step of the model, we can choose between different variables assessing the same physiologic concept, according to the specific monitoring tools used for a given patient. Nevertheless, this implies knowing exactly which amount of uncertainty each variable introduces into the model.

This model is limited by two mechanisms. First, conformance, a self-limiting oxygen requirement in case of tissue hypoxia may lead to a perception of an acceptable VO_2_ that may in fact be insufficient. Second, this model investigates only the macrocirculation. An acceptable global VO_2_ may hide important tissue heterogeneity. However, a conformance can be suspected by repeated measurements which is the purpose of a continuous monitoring and even though stabilizing the macro circulation is not the last word in haemodynamics, it is a prerequisite. Microcirculation cannot be optimized without stabilizing the macro circulation first. Therefore a model based on global tissue hypoxia is a sound basis for macrohemodynamic monitoring.

Other decision trees have been suggested that may be considered as suitable models for haemodynamic monitoring. However, none of them have been clinically validated and most of them have been developed from one or several variables proposed by industry to optimise the usefulness of a specific device [7]. A validated model strictly based on physiological knowledge, independent of actual technical issues, like the one we developed, is more likely to highlight the actual limitations and to help instigate the appropriate research.

### 2.2. What Are the Needed and Necessary Input/Output Variables for Describing This Model and Detecting Deterioration?

Once a global model has been described, the input and output variables are then clearly identified. Consensus on the definition of these terms is vital, otherwise, the reproducibility of the therapeutic actions based on the model would be poor. 

However, due to various reasons, including the choice of monitoring and/or actual technological limitations, one or several needed input variables may be unknown, or lacking, or assessed discontinuously. In these situations, it is important to still fit as closely as possible with the model despite the bias introduced by the missing input data. There are theoretically three solutions. First, it is sometimes possible to find a surrogate of a given input variable assuming that the information provided is close. This is the case for example replacing SvO_2_ by the central venous O_2_ saturation (ScVO_2_) or SaO_2_ by the pulse oximetry (SpO_2_) or total peripheral resistance instead of SVR when right aerial pressure is missing. Second, it is possible to give a fixed value to a given variable. For example, we may enter in the model directly at the level shown in Figure 2, assuming that the needed SV is the normal SV as a function of age and gender since the adaptative mechanisms of CO to needs are mostly resulting from a regulation of the heart rate. Third, it is possible to estimate a continuous variable from discontinuous clinical or paraclinical analysis. For example, VO_2_ is often not monitored and VO_2_ needs unknown. Consequently VO_2_/^*n*^VO_2_ ratio <1 is suspected by the presence of clinical signs of shock or by an increase in blood lactate over time. Alternatively, a change in VO_2_ needs (therefore translated to CO and EO_2_ needs) can, be estimated by age, gender, body size, body temperature and/or empiric estimation of the change in metabolic needs due to underlying or associated pathologies. In any case, if we restrict our analysis to a part of the global model, we must assume that the blind part is fixed and given an approximate value. The reliability of the model is therefore reduced.

### 2.3. What Are the Specific Quality Criteria and Tolerance for Each Input Variable?

The quality criteria and tolerance for the CO monitoring have been reviewed recently. [8, 9] The same effort must be done for each input variable of the model. Basically these criteria are very similar for all quantitative variables.

1. The accuracy is how close the value is to a gold standard. It is estimated by the mean difference (bias) with the true value given by the gold standard.
2. The linearity is the capability of maintaining constant the ratio between the physiologic signal and the electric output signal. Therefore the bias is constant. It can be verified by comparing the regression curve of the bias with the identity line.
3. The precision is the ability to indicate the same value when the physiologic signal is stable. In other words, it is the variation due to random error in the signal processing. It can be estimated by the standard deviation/mean value when the physiologic signal is stable. The least minimum significant change (smallest change indicating a real change) is a direct consequence of precision.
4. The resolution is the smallest change that the device can detect.
5. The stability is the capability of maintaining the preceding quality criteria unchanged during time (without drift).
6. The measuring range is the boundaries of value where the preceding quality criteria are found acceptable
7. The responsiveness is the delay between a real change in the physiologic signal and a change greater than the least minimum significant change in the observed value. Coupled with the linearity, it determines the accuracy of the amplitude response.

The six first quality criteria are common to all measurement and monitoring tools. The seventh, time-dependent quality criteria, is specific to monitoring devices.

For each quality criteria, it is important to determine the tolerance, that is, what is the allowed amount of variation from the exact value. Ideally, tolerance must be determined from clinical requirements, dependent on the way the variable is implemented in the model. However, we are obliged to consider the actual technological issues. Thus a 20% tolerance is seen as actually acceptable for CO while it is 4% for the haemoglobin concentration and 2% for the haemoglobin saturation. However, the accumulated effect of different tolerances may lead to poor performance. For example, a tolerance of 20% in accuracy for CO monitoring plus a tolerance of 5 minutes in time response, plus a tolerance of 10% in the linearity plus a tolerance of 10% in stability may result in the absence of detection of a transient 30% CO decrease. To study the impact of cumulated tolerances on a monitored variable, it can be useful to determine what is a clinically relevant change of this variable and to measure the sensitivity and the specificity of a given device to detect these clinically relevant changes.

### 2.4. Based on These Criteria, What Are the Validated Available Technologies for Monitoring Each Input Variable, Continuously, Real time, and If Possible NonInvasively?

Full validation of quality criteria below entails several considerations.

**Choice of the Patient Population**

A validation study must be applicable to all ICU patients. The haemodynamic profiles of sepsis and cardiac surgery are very different A useful device must provide accurate and precise data in all of these circumstances or extremes and this must be taken into account during the design of the validation studies. A monitoring device is also designed to detect and track changes in a variable over time. This can best be performed by building into the protocol an intervention that should on its own change the monitored variable.

**Choice of the Reference Method**

A monitoring system is typically a real-time, automatic, and continuous analyzer. An ideal device should provide good data from both snapshot measurements and continuous monitoring. Any validation study therefore needs to incorporate both of these factors into its design, especially with respect to its choice of a gold standard. A gold standard is most often lacking in clinical practice and we are obliged to consider simply a reference technology. However, few technologies even when they are considered as a reference method have fulfilled all the quality criteria listed above. Therefore, It may be necessary to choose a different reference method for studying separately each quality criteria.

**Data Acquisition Method**

A completely automatic continuous data recording technique should be preferably utilized for both the reference method and the studied technology in order to avoid errors when collecting large numbers of data points and also to limit any interobserver variability. It is preferable to collect raw data that has not been time-averaged by the device, in order to minimize the smart averaging and the smoothing effect used in many modern technologies. The rationale behind this is that the better the raw signal, the better the result will be after final averaging. As mathematically predicted, averaging more data decreases the variability but not the accuracy [10]. It must not be forgotten, however, that the devices will be ultimately used according to the data that they display, which quite often will be different to the raw numbers. How it affects the validation in the subsequent analysis must be explained and described in detail.

**Data Acceptability**

Before analysing the data collected in the fashion described above, it is important to validate the data. This requires an independent assessor who is blind to the choice of monitoring technology assessing the data. Periods of time when the patients are agitated, when one or both systems became disconnected, or periods of time where there is clear evidence of a situation leading to artefact can be deleted. This is a critical step of the validation that can be altered by subjective choices.

**Data Segmentation**

The monitoring trend line of a given variable can be schematically divided into periods of unchanged, increasing, or decreasing value. Fulfilling the criteria of quality determined above requires studying these different periods of time separately in order to minimize the physiological variability. Easy database segmentation can be performed using the trend line slope of the monitored variable. The inflexion point between two consecutive slopes may be automatically determined using the minimum sum of residuals for the two segments proposed by John-Alder [11].

Few technologies have been extensively studied using the quality criteria listed above [12]. We can consider that this has been achieved satisfactorily only for temperature and HR monitoring and for Hb, SaO_2_, SvO_2_, fand lactate measurements. Blood pressure monitoring (RAP, PAP, SAP) with actual transducers, appropriate lines, and filtering may be considered acceptable if properly maintained and positioned. SvO_2_ and ScvO_2_ monitoring using infrared spectroscopy may also be considered acceptable if properly positioned, flushed, and recalibrated.

All other monitoring variables have various degrees of limitations regarding the listed quality criteria, especially CO, SaO_2_, Hb, lactate, ventricle filling and contractility indices, tissue perfusion indices. However, a complete analysis of these imitations is a prerequisite before using these variables in intelligent hemodynamic monitoring. The cumulative effects of these limitations may lead to poor results.

### 2.5. How Can We Integrate All of the Needed Reliably Monitored Input Variables on the Same System for Continuously Describing the Hemodynamic Model?

Different companies currently develop integrating systems. But the reliability of the different variables provided by the same company may not be optimal. The best chance would be given by integrating a maximum number of reliable variables coming from different devices manufactured by different companies. It is a challenge for scientific societies such as the ESICM to encourage such developments.

### 2.6. Is It Possible to Implement This Reliably Described Haemodynamic Model into Intelligent Programs Able to Differentiate Significant Changes versus Artifactual Changes and to Display Intelligent Messages and/or Diagnosis?

This still requires a proper solution. An available intelligent program developed for analysing snapshot measurements [1, 2, 5] can be a basis for comparing trends. Including automatic trend lines in a reasoning process instead of validated measurements requires that artefacts be filtered out. It is also necessary to determine the time sampling to avoid continuous instantaneous diagnostic changes with no practical use. Once this will have been done, we will be ready to check if this type of monitoring is likely to increase the speed and appropriateness of therapeutic interventions and to improve outcome.

## 3. Conclusion

No matter how sophisticated and advanced the integrated monitoring system is in an intensive care unit, its ability to detect that a patient is deteriorating will still depend on the quality of the staff in the critical care unit and their ability to work as a team. More sophisticated monitoring should not be used at the expense of reducing staff/patient ratios but rather to enhance the ability of the staff to manage patients. The procurement of an appropriate clinical information system where there is none should also be high on the wish list to help collect the many direct and indirect variables of patient data and allow clinical decision support analysis. It is the task of industry to maximise the information provided by their technology. It is the task of the medical community to describe the ideal tool they need.
